# Double mutation for multiple endocrine neoplasia associated with congenital adrenal hyperplasia

**DOI:** 10.1530/EDM-24-0047

**Published:** 2025-06-06

**Authors:** Watrusy Lima de Oliveira, Eloilda Maria de Aguiar Silva, Carlos Eduardo de Melo Oliveira, Wellington Alves Filho, Maria Cecília Martins Costa, Renata Carvalho de Alencar, Carla Antoniana de Almeida Vieira, Ana Rosa Pinto Quidute

**Affiliations:** ^1^ Endocrinology Program, Federal University of Ceará (UFC), Fortaleza, Brazil; ^2^ UFC/FUNCAP, Federal University of Ceará (UFC), Fortaleza, Brazil; ^3^ Head and Neck Surgery Service, HUWC/UFC/EBSERH, Federal University of Ceará (UFC), Fortaleza, Brazil; ^4^ Endocrinology Service, General Hospital of Fortaleza (HGF), Fortaleza, Brazil; ^5^ Endocrinology Service, HUWC/UFC/EBSERH, Federal University of Ceará (UFC), Fortaleza, Brazil; ^6^ Endocrinology and Metabology Program, HUWC/UFC/EBSERH, Federal University of Ceará (UFC), Fortaleza, Brazil; ^7^ Department of Physiology and Pharmacology, Drug Research and Development Center (NPDM), Faculty of Medicine, Federal University of Ceará (UFC), Fortaleza, Brazil

**Keywords:** multiple endocrine neoplasia type 1, multiple endocrine neoplasia type 2, adrenal hyperplasia, congenital

## Abstract

**Summary:**

A 39 year old female with signs of hyperandrogenism, was diagnosed with congenital adrenal hyperplasia after a cortrosyn test. Abdominal tomography showed a nodular image in the right adrenal gland, measuring 1.9 × 3.1 cm, 26 UH. Screening for Cushing’s syndrome and pheochromocytoma was negative. Due to the maternal family history of *MEN2A*, *RET* gene testing was performed (positive), and screening for medullary thyroid carcinoma (MTC) with calcitonin was <2 pg/mL. As the patient’s father passed away due to complications of peptic ulcers and hailed from a region with high rates of MEN1, PTH and calcium levels were checked, confirming the diagnosis of primary hyperparathyroidism (pHPT). Genetic investigation for *MEN1* was positive, and an MRI of the pituitary gland revealed a non-producing macroadenoma. Management of pHPT and total prophylactic thyroidectomy were recommended, with an expectant approach regarding the adrenal lesion. Histopathological examination of the thyroid revealed papillary microcarcinoma in the right lobe and parafollicular cell hyperplasia in two foci, with immunohistochemistry consistent with MTC.

**Learning points:**

- There may be the coexistence of three rare and complex genetic syndromes in a single patient.
- Multiple endocrine neoplasia syndromes describe a group of heterogeneous diseases.
- Hyperparathyroidism is common among multiple endocrine neoplasia syndromes.

## Background

Multiple endocrine neoplasias (*MEN*) are complex genetic syndromes, comprising four recognized disorders (*MEN 1–4*) that are autosomal dominant and are phenotypically distinguished by the development of synchronous or metachronous tumors in specific endocrine glands (1).

*MEN1* is characterized by variable combinations of more than 20 endocrine and non-endocrine tumors associated with loss of heterozygosity (LOH) at 11q13, the location of the *MEN1* gene, resulting in biallelic loss of *MEN1*. Endocrine tumors become evident due to hormonal overproduction or tumor growth. Diagnosis is clinically suspected by the occurrence of two or more classic endocrinopathies: primary hyperparathyroidism (pHPT) due to parathyroid hyperplasia, anterior pituitary tumors and duodenopancreatic neuroendocrine tumors (DP-NETs). Other associated tumors include thymic and pulmonary NETs, type 2 gastric NETs, adrenocortical tumors, pheochromocytomas, facial angiofibromas, collagenomas, hibernomas, meningiomas, ependymomas, leiomyomas, lipomas and a higher risk of breast cancer in women. Rare neoplasms, such as carcinoid tumors, parathyroid carcinoma and adrenocortical carcinoma, are the leading causes of mortality in *MEN1* patients (2).

*MEN2* is attributed to a genetic defect in the proto-oncogene rearranged during transfection (*RET*) on chromosome 10, leading to a gain-of-function in the RET tyrosine kinase receptor. As a result, cell growth, proliferation and differentiation are promoted, leading to the formation of multiple tumors in all tissues where RET is predominantly expressed (thyroid C cells, adrenal medulla chromaffin cells, parathyroid chief and oxyphil cells, sympathetic, parasympathetic and enteric ganglia, and the urogenital tract) (3).

Congenital adrenal hyperplasia (CAH), in turn, is a genetic autosomal recessive disorder that impairs cortisol production due to a deficiency in one of the five essential enzymes for its biosynthesis. This deficiency lowers cortisol levels, weakening the negative feedback on the pituitary gland, which increases adrenocorticotropic hormone (ACTH), excessively stimulating the adrenal gland and causing hyperplasia of its cortex. The deficiency of 21-hydroxylase (21-OH) is the most common form of CAH, accounting for approximately 90–95% of cases. This condition leads to a reduction in cortisol biosynthesis and increased levels of ACTH, 17-hydroxyprogesterone (17-OHP) and adrenal androgens (4).

To date, there are no previous reports in the literature of the association between congenital adrenal hyperplasia and multiple endocrine neoplasia, and there is only one previously reported family showing manifestations of *MEN1* and *MEN2* with a germline mutation in the *RET* proto-oncogene and *MEN1* germline mutation detected.

## Case presentation

A 39 year old female was referred to the endocrinology service in 2018 with menstrual irregularity, hirsutism on the chin and root of the thighs, acne and androgenic alopecia. On physical examination, she presented a weight of 72.25 kg, BMI: 29.7, trichoepitheliomas and neurofibromas throughout the body. Laboratory evaluation showed hyperandrogenism and basal 17-alpha-OH-progesterone of 286 ng/dL. She underwent a cortrosyn test with 17-α-OH-progesterone of 1,767 ng/dL after stimulation, confirming the diagnosis of CAH.

**Figure 1:**
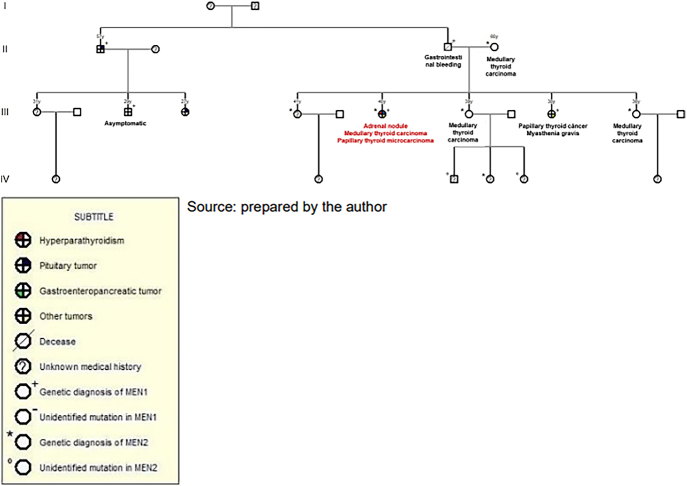
Pedigree diagram.

**Figure 2:**
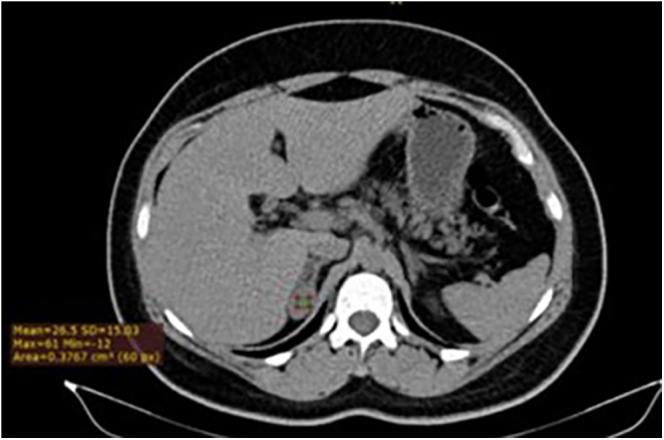
Abdominal CT scan without contrast demonstrates a nodular image in the right adrenal gland, measuring 1.9 × 3.1 cm and 26 HU.

Due to the maternal family history of *MEN2A* (Fig. 1), the patient underwent *RET* gene screening, resulting positive for the *p.Lys666Asn* mutation (c.1998G>C). An abdominal computed tomography (CT) scan (Fig. 2) revealed a nodular image in the right adrenal gland, measuring 1.9 × 3.1 cm, 26 UH, and, so far, she has had negative clinical and laboratory screenings for pheochromocytoma and Cushing’s syndrome. In addition, she had a negative initial screening for medullary thyroid carcinoma (MTC).

The patient’s father passed away due to complications from peptic ulcer and was originally from Baixo Jaguaribe, a microregion in Ceará with high rates of *MEN1*, according to data from a previous study that evaluated the oral health of a group of patients from the region with a familial form of *MEN1*, monitored at the Clinical Endocrinology and Diabetes department of the Hospital Complex of the Federal University of Ceará (CH-UFC/Ebserh) in Fortaleza, CE (5). Based on this background, we performed parathyroid hormone (PTH) and calcium tests, which confirmed the diagnosis of primary hyperparathyroidism.

**Figure 3:**
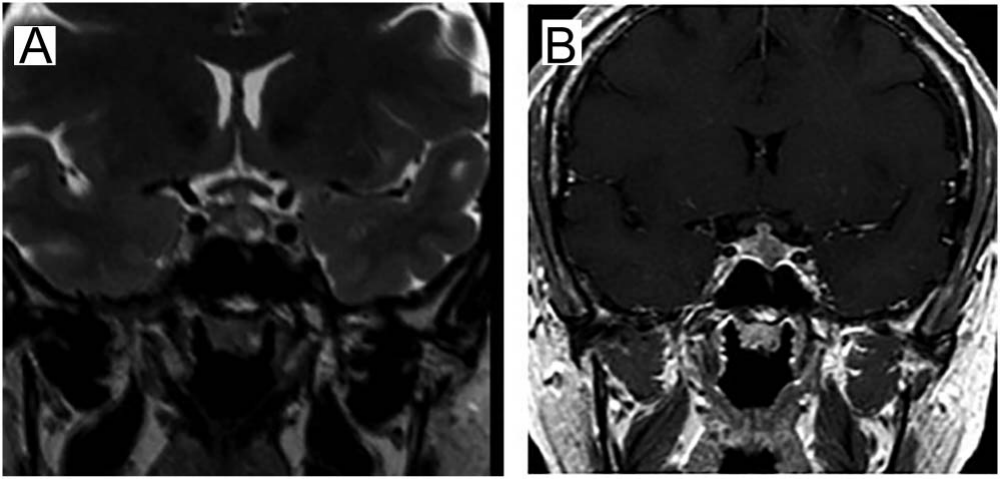
MRI of the sella turcica, coronal slices weighted in T2 (A) and post-contrast T1 (B), demonstrates an elongated formation measuring 1.0 × 0.5 cm, suggestive of a pituitary adenoma.

**Figure 4:**
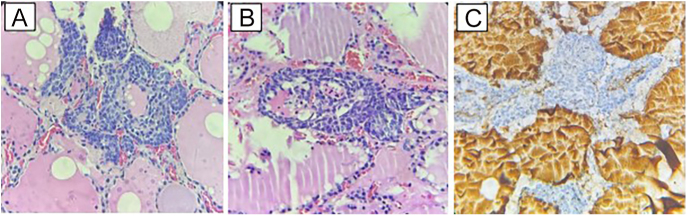
Medullary microcarcinoma in two foci: right lobe (HE-400×) (A), left lobe (HE-400×) (B). IHC for Tg negative in right lobe – 400× (C).

The patient was directed for genetic investigation of *MEN1*, presenting a pathogenic variant *IVS3 + G > T* in the *MEN1* gene. She underwent pituitary MRI (Fig. 3), revealing an elongated formation measuring 1.0 × 0.5 cm, suggestive of pituitary adenoma, with normal levels of IGF1, GH, prolactin and TSH. Given the clinical scenario, the proposed treatment for the patient was a unilateral surgical approach to the right parathyroids, also opting at this moment for total prophylactic thyroidectomy and expectant management regarding the non-functioning adrenal adenoma. Thyroid histopathology showed a classical subtype papillary microcarcinoma (PTMC) in the right lobe, measuring 1.8 × 1.6 mm (pT1a – low risk). In addition, hyperplasia of parafollicular cells was described in two foci (right and left lobes), with immunohistochemistry compatible with medullary thyroid microcarcinoma in both foci (positive chromogranin A and synaptophysin) (Fig. 4).

Currently, the patient is without evidence of recurrent PTMC and has controlled serum levels of PTH and calcium. She remains under monitoring for existing lesions and regular screening for the various associated pathologies. Supplementary exams performed are described in Tables 1 and 2.

**Table 1:** Laboratory tests.

Investigations	Result	Reference intervals
Laboratory tests		
Total testosterone, ng/dL	78.4	14–76
Unbound testosterone, ng/dL	2.22	0.18–1.68
DHEAS, μg/dL	361	45–270
17-hydroxyprogesterone, ng/dL	286	
SST for 17-hydroxyprogesterone, ng/dL	1,767	
Calcitonin, pg/mL	<2	
Urinary metanephrines, μg/24 h	521	<1,000
Cortisol (post 1 mg DEX), μg/dL	1.32	
PTH, pg/mL	447	11–67
Calcium, mg/dL	10.16	8.8–11
Albumin, g/dL	4	3.5–5.2
Albumin-corrected calcium, mg/dL	10.16	
25(OH)D, ng/mL	26.4	30–60
IGF1, ng/mL	215	107–246
GH, μg/L	0.06	
Prolactin, ng/mL	19.4	
Basal cortisol, μg/dL	8.5	
TSH, μIU/mL	2.38	0.4–4.0
Postoperative studies		
PTH, pg/mL	42.8	VR: 18.5–88
Calcium, mg/dL	9.3	VR: 8.3–10.6
TSH, μIU/mL	1.11	VR: 0.5–2

PTH, parathyroid hormone; IGF1, insulin-like growth factor-1; GH, growth hormone; TSH, thyroid stimulating hormone.

**Table 2:** Radiological results.

Imaging studies	Findings
Thyroid ultrasound (2019)	Normal
Abdominal CT with contrast (Fig. 2)	Nodular image in the right adrenal gland, density of 26 HU (1.9 × 3.1 cm), absolute washout: 54.1% and relative: 29.9%
MIBG scintigraphy	Normal
Thyroid ultrasound (2021)	Hypoechoic nodule measuring 2.2 × 1.5 × 0.8 cm adjacent to the lower pole of the right thyroid lobe, suggesting adenoma/parathyroid hyperplasia
Parathyroid scintigraphy	Focal hypercaptation in the projection in the lower portion of the right lobe
Abdominal CT without contrast	Bilateral non-obstructive renal microlithiasis
Bone mineral densitometry	Normal
Pituitary MRI (Fig. 3)	Expansive lesion in the left adenohypophysis with hypointense signal on T1 and heterogeneous signal on T2, showing decreased contrast enhancement

## Discussion

The *MEN* describe a heterogeneous group of disorders characterized by predisposition to tumors involving two or more endocrine glands (1). Although some tumors may manifest in both syndromes, the interaction between molecular pathways related to *MEN1* and *RET* gene products remains unclear. In addition, certain polymorphisms may influence disease course or result in novel phenotypic manifestations (6).

This patient presented with *MEN*, associating primary hyperparathyroidism, non-secretory macroadenoma, non-secretory adrenal tumor and MTC. Notably, the disease exhibited characteristics of both *MEN1* (non-secretory macroadenoma and adrenal tumor) and *MEN2* (MTC), while hyperparathyroidism is common to both syndromes.

Initial suspicion of *MEN2A* was raised due to positive maternal family history (Fig. 1) and confirmed by genetic screening. Pheochromocytoma and Cushing’s syndrome were ruled out and the adrenal lesion was monitored due to its stability on recent imaging studies. Only after prophylactic total thyroidectomy was MTC detected at an early stage.

MTC is the main characteristic of all *MEN2* subtypes and the most important determinant of mortality in these patients. Common mutations in the *RET* proto-oncogene guide the clinical management of *MEN2*, whereas rare or uncertain variants – such as *RETK666N*, the mutation identified in this patient – pose diagnostic and therapeutic challenges (7).

A 2010 study identified four novel germline *RET* variants in exons 8 and 11 that exhibited increased tyrosine kinase activity and *in vitro* transforming potential. Among these, the *p.K666N* variant (exon 11) stood out for its high tyrosine kinase activity and oncogenic potential (8).

Another study on the *RET K666N* mutation in a patient with MTC and bilateral pheochromocytoma (PHEO), who was homozygous for *RET K666N*, described important aspects of this mutation’s influence on the development of MTC and PHEOs in patients with *MEN2* syndrome. It indicated that individuals heterozygous for this mutation exhibit reduced penetrance for MTC without other *MEN2A* features. In addition, homozygosity for the mutation could contribute to the development of bilateral PHEO. Another relevant point is the clinical variability between homozygous and heterozygous individuals, suggesting that a second mutation may be necessary for full disease manifestation, indicating a gene dosage effect on *RET* activity (9).

Evidence therefore suggests an oncogenic potential for *RETK666N*, including its transforming activity in NIH3T3 cells and the association of other codon 666 mutations (*K666E, K666M, K666delinsNS and K666R*) with MTC. Nevertheless, the lack of robust familial studies limits definitive conclusions about its clinical role and further evaluations are essential to clarify its impact (7).

*MEN1* diagnosis was subsequently suspected due to widespread neurofibromas, paternal history suggestive of neuroendocrine tumors and the patient’s origin from a region with high disease prevalence. Further screening identified additional clinical characteristics. Hyperandrogenism, the initial presentation, can be associated with various conditions leading to excess androgen production by ovaries or adrenal glands. The most common cause of hyperandrogenism is polycystic ovary syndrome (PCOS), characterized by hyperandrogenism and chronic anovulation. Other causes, including congenital adrenal hyperplasia (CAH), must be excluded. The patient’s non-classical 21-hydroxylase deficiency (21OHD) presented with hyperandrogenic manifestations from late childhood to early adulthood (4).

To date, no previous reports associate CAH with multiple endocrine neoplasia. Only one family with *MEN1* and *MEN2* manifestations, harboring germline mutations in both *RET* and *MEN1* genes, has been reported (10). In this family, as in the present case, coexistence of *MEN1* and *RET* mutations did not alter tumor onset, behavior or typical phenotype compared to patients with isolated mutations.

## Declaration of interest

The authors declare that there is no conflict of interest that could be perceived as prejudicing the impartiality of the work reported.

## Funding

The study did not receive any specific grant from any funding agency in the public, commercial or not-for-profit sector.

## Author contribution statement

A R P Q contributed to formal analysis, project administration, supervision and writing review and editing. C A de A V and R C de A contributed to supervision and writing review and editing. E M de A S and C E de M O contributed to data curation, investigation and review and editing. M C M C and W A F contributed to writing review and editing. W L de O contributed to data curation, investigation, methodology, writing original draft and writing review and editing. 

## Patient consent

Written informed consent was obtained from the patient for publication of the submitted article and accompanying images.
